# Decorating the surface of *Escherichia coli* with bacterial lipoproteins: a comparative analysis of different display systems

**DOI:** 10.1186/s12934-021-01528-z

**Published:** 2021-02-02

**Authors:** Sonia Nicchi, Maria Giuliani, Fabiola Giusti, Laura Pancotto, Domenico Maione, Isabel Delany, Cesira L. Galeotti, Cecilia Brettoni

**Affiliations:** 1 GSK, via Fiorentina 1, 53100 Siena, Italy; 2 Department of Pharmacy and Biotechnology (FaBiT), University of Bologna, Bologna, Italy

**Keywords:** Surface display systems, Lpp’OmpA chimaera, Ice nucleation protein (InaK-NC), AIDA-I, *Escherichia coli*, Lipoproteins

## Abstract

**Background:**

The display of recombinant proteins on cell surfaces has a plethora of applications including vaccine development, screening of peptide libraries, whole-cell biocatalysts and biosensor development for diagnostic, industrial or environmental purposes. In the last decades, a wide variety of surface display systems have been developed for the exposure of recombinant proteins on the surface of *Escherichia coli*, such as autotransporters and outer membrane proteins.

**Results:**

In this study, we assess three approaches for the surface display of a panel of heterologous and homologous mature lipoproteins in *E. coli*: four from *Neisseria meningitidis* and four from the host strain that are known to be localised in the inner leaflet of the outer membrane. Constructs were made carrying the sequences coding for eight mature lipoproteins, each fused to the delivery portion of three different systems: the autotransporter adhesin involved in diffuse adherence-I (AIDA-I) from enteropathogenic *E. coli*, the Lpp’OmpA chimaera and a truncated form of the ice nucleation protein (INP), InaK-NC (N-terminal domain fused with C-terminal one) from *Pseudomonas syringae.* In contrast to what was observed for the INP constructs, when fused to the AIDA-I or Lpp’OmpA, most of the mature lipoproteins were displayed on the bacterial surface both at 37 and 25 °C as demonstrated by FACS analysis, confocal and transmission electron microscopy.

**Conclusions:**

To our knowledge this is the first study that compares surface display systems using a number of passenger proteins. We have shown that the experimental conditions, including the choice of the carrier protein and the growth temperature, play an important role in the translocation of mature lipoproteins onto the bacterial surface*.* Despite all the optimization steps performed with the InaK-NC anchor motif, surface exposure of the passenger proteins used in this study was not achieved. For our experimental conditions, Lpp’OmpA chimaera has proved to be an efficient surface display system for the homologous passenger proteins although cell lysis and phenotype heterogeneity were observed. Finally, AIDA-I was found to be the best surface display system for mature lipoproteins (especially heterologous ones) in the *E. coli* host strain with no inhibition of growth and only limited phenotype heterogeneity.

## Background

Yeast [1, 2] mammalian [3, 4], insect [5, 6] and bacterial cells [7, 8, 9] have been used to display recombinant proteins on their cell surfaces for variou, cell-surface display applications including vaccine development, screening of peptide libraries, whole-cell biocatalysts and biosensor development for diagnostic, industrial or environmental purposes [10, 11]. Among bacterial host strains, *Escherichia coli* is the most widely used as it is genetically well-characterised and has extraordinary versatility due to its rapid growth and ease of genetic manipulation [12]. Over the past years, autotransporters and outer membrane proteins have been efficiently exploited as carrier proteins for the exposure of recombinant proteins (passenger proteins) on the surface of *E. coli* [13]. Among them, the adhesin involved in diffuse adherence-I (AIDA-I) from enteropathogenic *E. coli* (EPEC) strains [14, 15, 16] the Lpp’OmpA chimaera [17, 18, 19] and the ice nucleation protein (INP) from *Pseudomonas syringae* [20, 21, 22] have been successfully used as targeting vehicles for localising a great number of prokaryotic and eukaryotic full-length soluble proteins, protein domains or peptides on the surface of *E. coli* [13].

These three carrier proteins exploit different mechanisms of translocation to the bacterial surface. AIDA-I is a monomeric autotransporter belonging to the Type V secretion system (TVSS) consisting of different functional domains [23]: an N-terminal signal peptide, a passenger domain harbouring biological activity in the extracellular space, a linker domain and a translocator domain which is predicted to form a β-barrel structure integrated into the outer membrane (OM) where it forms a pore through which the translocation of the passenger domain occurs [24]. Autotransporters were originally thought to be self-sufficient for secretion. However, several lines of evidence now strongly suggest that both the secretion of the passenger domain and the membrane integration of the β barrel domain are catalysed by the barrel assembly machinery (BAM) complex and perhaps by an additional complex, named translocation and assembly module or TAM [25, 26]. It has been proposed that the TAM complex may either boost the activity of the BAM complex (consecutive role or by simultaneous cooperation), or function as a backup translocase activated only under high secretory demand [27]. The AIDA-I secretion mechanism can be exploited for the surface exposure of recombinant proteins in *E. coli* by simply replacing the coding region of the natural passenger domain (N-terminus) with that of the recombinant protein of interest [28].

Lpp’OmpA is a chimaera developed by Georgiou and co-workers consisting of the signal peptide and the first nine residues of Braun’s lipoprotein or Lpp (Lpp’), responsible for the targeting to the outer membrane, fused with five of the eight membrane-spanning segments of the OmpA porin (residues 46–159). In this case, the protein of interest is fused at the C-terminus of Lpp’OmpA [29]. Although the exact mechanism of translocation exploited by this chimaera has never been described in detail, we can speculate that it entails a combination of lipoprotein and outer membrane protein translocation mechanisms. After the export to the inner membrane (IM), the cysteine residue of Lpp’ undergoes lipid modifications in a sequential process catalysed by three periplasmic enzymes: diacylglyceryl transferase (Lgt), signal peptidase II (LspA) and N-acyltransferase (Lnt) [30]. Subsequently, translocation to the OM is determined by the identity of the amino acids that follow the conserved cysteine, and this leads to the recognition by the localisation of lipoproteins (Lol) pathway [31, 32]. In addition, the correct insertion of the five membrane-spanning segments of the OmpA porin may require the action of the BAM complex [33].

The last delivery system analysed is the ice nucleation protein (INP) of *Pseudomonas syringae*. INP is an outer membrane protein that is found in several plant pathogenic bacteria [34]. In *P. syringae,* InaK, a member of the INP family, is characterised by the presence of three domains. The N-domain is relatively hydrophobic and seems to be the only domain responsible for the targeting to the cell surface. An exposed central part called central repeated domain (CRD) comprises a series of contiguous repeats that act as a template for ice crystal formation. The C-terminal domain is hydrophilic and exposed to the extracellular environment [35]. INP is attached to the outer cell membrane via a glycosylphosphatidylinositol (GPI) anchor in a manner similar to that observed in eukaryotic cells. In addition, three asparagine residues in the N-terminus and one conserved threonine residue in the C-terminus enable the protein to be coupled to various sugars through N- and O-glycan linkages [35]. It has been shown that full-length INP and various truncated forms yield stable surface display [13]. The construct used in this work comprises only the N-terminal domain fused to C-terminal domains without CRD (InaK-NC) and allows C-terminal fusion of the protein of interest.

All three delivery systems have been shown to efficiently expose heterologous proteins on the surface of *E. coli* [13], however, their direct comparison has not been previously reported. For the first time, these three approaches are assessed for the surface exposure of several passenger proteins belonging to the same class, the mature portion of lipoproteins. Full-length lipoproteins constitute a specific class of membrane proteins that have been shown to be potential vaccine candidates as they play key roles in adhesion to host cells, modulation of inflammatory processes and translocation of virulence factors into host cells [36, 37]. In this study, a panel of eight lipoproteins was considered: four from *Neisseria meningitidis* and four from the host strain that are known to be localised in the inner leaflet of the outer membrane. The expression of the resulting thirty-two constructs comprising the eight full-length lipoproteins and their mature portion fused to AIDA-I, Lpp’OmpA and InaK-NC was enabled under the control of the T7 promoter. A number of approaches were used to investigate their localisation on the surface of bacterial cells: FACS analysis, confocal and transmission electron microscopy.

## Results

### Delivery systems engineering: AIDA-I, Lpp’OmpA and InaK-NC

**Fig. 1:**
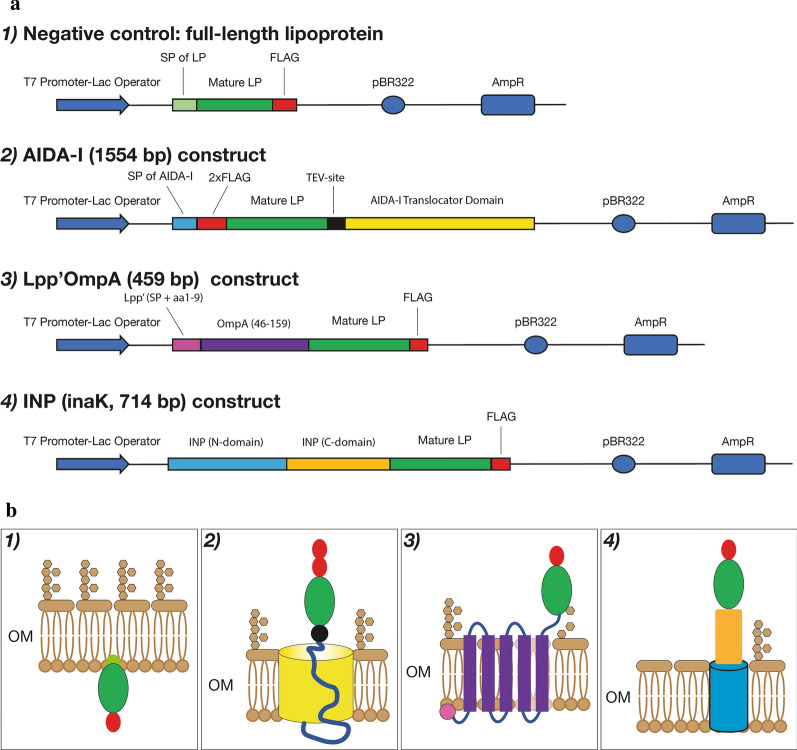
Schematic representation of the expression and display delivery systems. **a** Schematic representation of the domains of the constructs expressing recombinant versions of the eight proteins in the four different genetically engineered systems. **b** The panel illustrates schematically the display on the OM of each construct including 1) the full-length lipoprotein with its own signal peptide including lipobox lacking a carrier protein system and the mature portion of the lipoprotein fused to 2) AIDA-I, 3) Lpp’OmpA and 4) INP**,** InaK (N-domain + C-domain). In both panels (**a** and **b**), each protein domain is displayed with different colours. *SP* signal peptide, *LP* lipoprotein. *OM* outer membrane

A total of thirty-two constructs comprising the eight full-length lipoproteins and their mature portions fused to AIDA-I, Lpp’OmpA or InaK-NC were engineered in the pET15b expression plasmid as follows (Fig. 1):

1. As negative controls, the eight full-length lipoproteins retaining their own signal peptide were cloned in the absence of a carrier delivery system.
2. Each construct with AIDA-I (1554 bp) as a delivery system consists of: an N-terminal signal peptide, the FLAG tag, the TEV (Tobacco Etch Virus) protease cleavage site, a flexible linker and the AIDA-I translocator unit. The sequence of each mature lipoprotein was cloned between the FLAG and the TEV cleavage site.
3. Each Lpp’OmpA fusion (454 bp) consists of the signal peptide sequence, the first nine residues of Lpp (this region is indicated as Lpp’) and residues 46–159 of OmpA comprising five of the eight membrane-spanning segments found in the native protein. The FLAG tag is located at the C-terminus of these constructs. The sequence coding for each mature lipoprotein was cloned between the last membrane-spanning segment of OmpA and the FLAG tag.
4. Each construct of the ice nucleation protein (714 bp) comprises the N-terminal and C-terminal domains of the protein, but lacks all of the central repeating domain (InaK-NC). The FLAG tag is located at the C-terminus of each construct. The sequence of each mature lipoprotein was cloned between the C-terminal domain of INP and the FLAG.

The panel of the heterologous lipoproteins analysed comprises CsgG, MtrC, BamE and a putative lipoprotein from *Neisseria meningitidis* (NZ98/254) for which there are no data concerning their behaviour in *E. coli* as a host strain. In addition, four lipoproteins from *E.coli* K-12 were also analysed: Pal [38], BamE [33], LptE [39] and LolB [40] that are known to be localised in the inner leaflet of the outer membrane. Hence, they represent a useful control to evaluate the efficiency of the three delivery systems. The structure and description of their function are reported in Table 1. These lipoproteins have a molecular mass ranging from 10 to 40 kDa. The percentage of secondary structures present in each lipoprotein was investigated by using the SOPMA software (https://npsa-prabi.ibcp.fr/cgi-bin/npsa_automat.pl?page=/NPSA/npsa_sopma.html). As can be deduced from Table 1, all the lipoproteins under study have a similar content of alpha helix, beta strand, beta turn and random coils. Hence, only the size and the origin (homologous or heterologous) of the passenger proteins may have an influence on the process of surface translocation.

**Table 1:** Description of the passenger proteins expressed in this study

Lipoprotein	MW (kDa)	Function	Alpha helix %	Beta strand %	Beta turn %	Random coil %
*Nm*-CsgG	21.75	Involved in curli production, amyloid fibre associated with biofilm formation, host cell adhesion and invasion	44.39	18.39	4.48	32.74
*Nm*-MtrC	40.33	Belongs to the *mtr* gene complex, encodes an efflux pump system responsible for Cationic Antimicrobial Peptide Resistance	33.01	20.63	8.98	37.38
*Nm*-BamE	12.14	Homologue to *E. coli* BamE	32.8	18.4	8.8	40
Putative Lipoprotein	11.09	Hypothetical lipoprotein	51.22	6.5	8.94	33.33
*Ec*-Pal	16.68	Belongs to the Tol-Pal system. Plays a role in outer membrane invagination during cell division and outer membrane integrity	41.04	15.03	5.78	38.15
*Ec-*BamE	9.98	Modulates the conformation of BamA (lateral opening). Key role in the OMP assembly process and cell envelope conformation	25.66	24.78	7.08	42.48
*Ec-*LptE	18.86	Involved in the insertion of LPS into the OM, facilitating O-antigen translocation. Mostly nested in the β-barrel lumen of LptD	39.41	18.24	4.71	37.65
*Ec*-LolB	21.04	Essential outer membrane lipoprotein, accepts lipoproteins from LolA, mediates the outer membrane anchoring of lipoproteins	21.26	26.09	6.28	46.38

### Surface display of bacterial lipoproteins in *E. coli* evaluated by FACS analysis

**Fig. 2:**
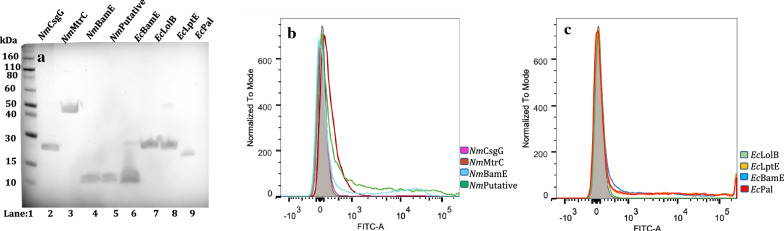
Expression and surface display of the full-length lipoproteins in *E. coli,* at 37 °C.** a** Western blot of whole-cell lysates showing full-length lipoproteins. Lane 1: Marker, Lane 2: *Nm*CsgG, Lane 3: *Nm*MtrC, Lane 4: *Nm*BamE, Lane 5: *Nm* putative lipoprotein, Lane 6: *Ec*BamE, Lane 7: *Ec*LolB, Lane 8: *Ec*LptE and Lane 9: *Ec*Pal. FLAG-tag specific antibodies were used for detection. **b**, **c** FACS analysis of *E. coli* expressing full-length lipoproteins at 37 °C. At 37 °C, *E. coli* BL21DE3 (pET15b) expressing *N. meningitidis* lipoproteins: CsgG, MtrC, BamE and a putative lipoprotein (**b**) and *E. coli* lipoproteins (**c**)*:* BamE, LolB, LptE and Pal were incubated with the monoclonal anti-FLAG antibody. The grey areas represent the fluorescence signals obtained with the control (BL21DE3-pET15b ∅). The coloured lines represent the full-length lipoproteins. Panel B (heterologous lipoproteins): Purple: CsgG, Dark Red: MtrC, Light Blue: nmBamE, Dark Green: putative lipoprotein; Panel C (homologous lipoproteins): Dark Blue: BamE, Light Green: LolB, Orange: LptE, Light Red: Pal

**Fig. 3:**
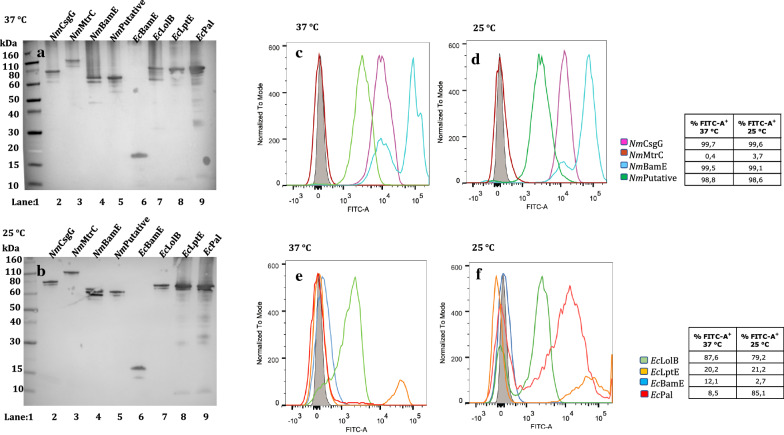
Expression and surface display of AIDA-I fusion proteins in *E. coli,* at 37 and 25 °C. Western blot of whole-cell lysates showing AIDA-I fusions proteins. Lane 1: Marker, Lane 2: *Nm*CsgG, Lane 3: *Nm*MtrC, Lane 4: *Nm*BamE, Lane 5: *Nm* putative lipoprotein, Lane 6: *Ec*BamE, Lane 7: *Ec*LolB, Lane 8: *Ec*LptE and Lane 9: *Ec*Pal, at 37 °C (**a**) and at 25 °C (**b**). FLAG-tag specific antibodies were used for detection. FACS analysis of *E. coli* BL21DE3 (pET15b) expressing AIDA-I fused to the *N. meningitidis* lipoproteins: CsgG, MtrC, BamE and putative lipoprotein at 37 °C (**c**) and 25 °C (**d**) and the *E. coli* lipoproteins*:* LolB, LptE, Pal and BamE at 37 °C (**e**) and 25 °C (**f**) were incubated with monoclonal anti-FLAG antibody. The grey areas represent the fluorescence signals obtained with the control (BL21DE3-pET15b ∅). The coloured lines represent the fused forms of the lipoproteins. Panels C and D (heterologous lipoproteins): Purple: CsgG, Dark Red: MtrC, Light Blue: nmBamE, Dark Green: putative lipoprotein; Panels E and F (homologous lipoproteins): Dark Blue: BamE, Light Green: LolB, Orange: LptE, Light Red: Pal

**Fig. 4:**
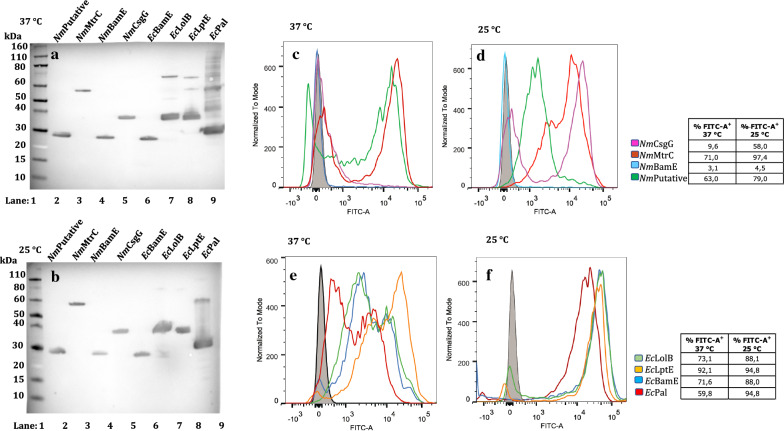
Expression and surface display of Lpp’OmpA fusion proteins in *E. coli,* at 37 and 25 °C. Western blot of whole-cell lysates showing Lpp’OmpA fusions proteins. Lane 1: Marker, Lane 2: *Nm* putative lipoprotein, Lane 3: *Nm*MtrC, Lane 4: *Nm*BamE, Lane 5: *Nm*CsgG, Lane 6: *Ec*BamE, Lane 7: *Ec*LolB, Lane 8: *Ec*LptE and Lane 9: *Ec*Pal, at 37 °C (**a**) and at 25 °C (**b**). FLAG-tag specific antibodies were used for detection. FACS analysis of *E. coli* BL21DE3 (pET15b) expressing Lpp’OmpA fused the *N. meningitidis* lipoproteins: CsgG, MtrC, BamE and putative lipoprotein, at 37 °C (**c**) and 25 °C (**d**) and the *E. coli* lipoproteins*:* LolB, LptE, Pal and BamE at 37 °C (**e**) and 25 °C (**f**) were incubated with monoclonal anti-FLAG antibody. The grey areas represent the fluorescence signals obtained with the control (BL21DE3-pET15b ∅). The coloured lines represent the fused forms of the lipoproteins. Panels C and D (heterologous lipoproteins): Purple: CsgG, Dark Red: MtrC, Light Blue: nmBamE, Dark Green: putative lipoprotein; Panels E and F (homologous lipoproteins): Dark Blue: BamE, Light Green: LolB, Orange: LptE, Light Red: Pal

**Fig. 5:**
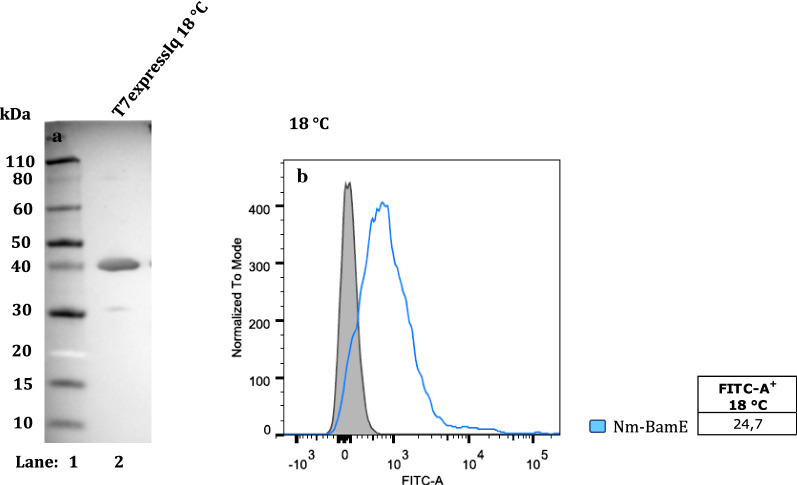
Expression and surface display of InaK fusion proteins in *E. coli,* at 18 °C. Western blot of whole-cell lysates showing InaK fusions proteins. Lane 1: Marker; Lane 2: T7ExpressIq expressing InaK-*Nm*BamE,at 18 °C (**a**). FACS analysis of *E. coli* T7ExpressIq (pET15b) expressing InaK fused the *N. meningitidis* lipoprotein BamE at 18 °C (**b**) was incubated with polyclonal anti-*Nm*BamE antibodies. The grey area represents the fluorescence signal obtained with the control (T7ExpressIq pET15b ∅). The light blue coloured line represents the fused form of the *Nm*BamE lipoprotein

All thirty-two constructs were introduced into *E. coli* and expression of the proteins was induced under growth conditions at various temperatures. The integrity and size of the different fusion proteins expressed were verified by Western blot and their surface exposure by FACS analysis. The results from Western blot and FACS experiments, from each of the four different genetically engineered systems, are reported in Figs. 2, 3, 4, 5. In order to simplify the interpretation for every given experimental condition, the lipoproteins are shown in two different panels according to their homologous (*E. coli*) or heterologous (*N. meningitidis*) origin. The relative percentage of positive cell populations (FITC-A +) is reported in the figure legends.

Both the neisserial and *E. coli* full-length lipoproteins were well-expressed (Fig. 2, panel A) and as shown by the FACs analysis, in which all of the eight different coloured traces perfectly overlap with the negative control, none of the proteins were surface-exposed at a growth temperature of 37 °C (Fig. 2, panels B and C, respectively) nor at 25 °C (data not shown).

When fused to AIDA-I, all mature lipoprotein domains were well-expressed both at 37 and 25 °C with the exception of *E.coli* BamE for which a low molecular weight band was present. This does not correspond to the form fused with AIDA-I and it may actually represent a cleaved form, suggesting that this protein undergoes proteolysis upon expression (Fig. 3 panels A and B). Three of the four neisserial lipoproteins (CsgG, BamE and putative lipoprotein) were displayed on the bacterial cell surface at both growth temperatures, 37 and 25 °C (Fig. 3, panels C and D), with a positive fluorescence signal that was significantly shifted for *Nm*BamE. The lipoprotein with the highest molecular weight, MtrC, was not surface-exposed at either growth temperature (Fig. 3, panels C and D). In the case of the *E. coli* lipoproteins (Fig. 3, panels E and F), LolB was exposed on the surface of *E. coli* at both temperatures, while the Pal and LptE AIDA-fusions resulted in *E. coli* showing two populations either expressing or not the specific genes on the bacterial surface. The Pal lipoprotein was not surface exposed at 37 °C, but a significant sub-population gave a positive fluorescent peak at 25 °C. The LptE passenger protein of *E. coli* also showed the same two-population behaviour, but with a clearly lower percentage of cells expressing this protein on the bacterial cell surface compared to Pal at both temperatures. This behaviour may be due to bistability, a condition in which cells with the same genotype separate into two distinct phenotypic populations, for example either expressing or not a specific gene. The occurrence of bacterial subpopulations showing different phenotypic traits within the same culture are known to be often due to the insurgence of epigenetic events occurring in response to stress conditions [41]. In particular, in the present case it may be related to expression levels significantly higher than those characteristic of physiological conditions. This can lead to an overloading of the folding machinery and the trafficking systems, thus preventing the surface translocation or alternatively leading to a misfolded population on the surface causing the lack of the FLAG-tag recognition by the specific antibody [42, 43, 44, 45]. An intriguing aspect that is worth noting is that the percentage of viable and not aggregated bacterial cells accounts for up to 93% of the population (Additional file 4: Figure S1, panel A), indicating that AIDA-I constructs do not affect viability of the host strain.

All mature lipoprotein domains were expressed when fused to the Lpp’OmpA chimaera both at 37 and 25 °C (Fig. 4 panels A and B), whereas the surface exposure of the proteins displayed some variability. Bacteria overexpressing these constructs showed a remarkable heterogeneity at 37 °C. This could be deduced by the broad distribution of the fluorescence intensity and by the presence of two distinct positive fluorescence signals (Fig. 4, panels C and E), indicating populations expressing variable amounts of protein on the surface. Lower heterogeneity was observed when the growth temperature was reduced to 25 °C, which may reflect a better coordination between rate of translation and secretion (Fig. 4, panels D and F). In the case of the neisserial lipoproteins (Fig. 4, panels C and D), Lpp’OmpA-*Nm*BamE was not surface exposed under any experimental conditions. At 37 °C, for Lpp’OmpA fusions with MtrC and the putative lipoprotein, two populations were detected either expressing or not the specific genes on the bacterial surface, but upon lowering the growth temperature to 25 °C, the negative population completely disappeared either through increased surface expression or decreased misfolding at 25 °C. CsgG fused to the Lpp’OmpA chimaera was not surface exposed at 37 °C but was at 25 °C, although two populations were still present. By contrast, all the *E. coli* lipoproteins were surface exposed exhibiting distinct highly positive populations particularly at 25 °C. The main drawback to *E. coli* overexpressing Lpp’OmpA constructs is that, in most cases, viable and not aggregated bacterial cells represent only 64% of the population (Additional file 4: Figure S1, panel B).

In the case of the InaK carrier protein, several experimental conditions including, growth temperature, concentration of inducer, time of induction and host strain selection have been investigated. Although the total cell extract revealed that constructs with INP were expressed at high levels, as can be deduced by the presence of clearly visible bands in SDS-PAGE (Additional file 5: Figure S2 panel A), the FACS analysis indicated that none of the lipoproteins were surface-exposed at either 37 or 25 °C (Additional file 5: Figure S2, panels B-C and D-E, respectively).

One possible explanation for these results is that the chimeras are misfolded. To exclude that the negative fluorescence signals could be due to the lack of FLAG-tag exposure on the bacterial surface, the FACS experiments were repeated at 18 °C to improve folding however all constructs gave negative FACS results also at this temperature (data not shown). In addition, to confirm that the negative results were not due to lack of exposure of the FLAG-tag on the bacterial surface, we used polyclonal antibodies produced in mouse and raised against the neisserial BamE. Nevertheless, at 25 °C the *Nm*BamE lipoprotein was not detectable also using the specific polyclonal antibody (Additional file 6: Figure S3, panel A). Since one important condition that can influence the folding of a chimaera is its level of expression, a different genetic background of the host strain T7express Iq (a BL21 *E. coli* derivative characterised by a mutation in the LacI gene that results in a reduced level of basal expression) was chosen. Even with this strain at 25 °C the fluorescence signal was still negative (Additional file 6: Figure S3, panel B). A partially positive FACS signal (24.7%) was obtained only in the case of the T7express Iq strain at 18 °C (Fig. 5, panel B). In WB analysis, the presence of a band corresponding to the molecular weight of the fusion protein indicated that it is well expressed even at this lower growth temperature (Fig. 5, panel A).

In summary, for our experimental conditions AIDA-I and Lpp’OmpA were the best delivery systems for the surface translocation of bacterial mature lipoproteins, with 25 °C representing the more favourable growth temperature to obtain a homogenous population of bacterial cells expressing the passengers of interest on the bacterial surface. Despite the fact that INP is considered one of the most promising carrier proteins, modulation of many experimental conditions for the InaK-NC construct was not enough to achieve levels of surface exposure comparable to those observed for the other two delivery systems.

### Insights into the surface localisation of passenger protein BamE

In addition to the FACS experiments, the surface localisation of the four different genetically engineered systems of BamE passenger protein was observed by using confocal microscopy and transmission electron microscopy. To this end, as a representative example, we used the anti-BamE polyclonal serum for the INP construct (the only experimental condition that gave us positive signals in the FACS analysis) and the FLAG-antibodies for the others.

**Fig. 6:**
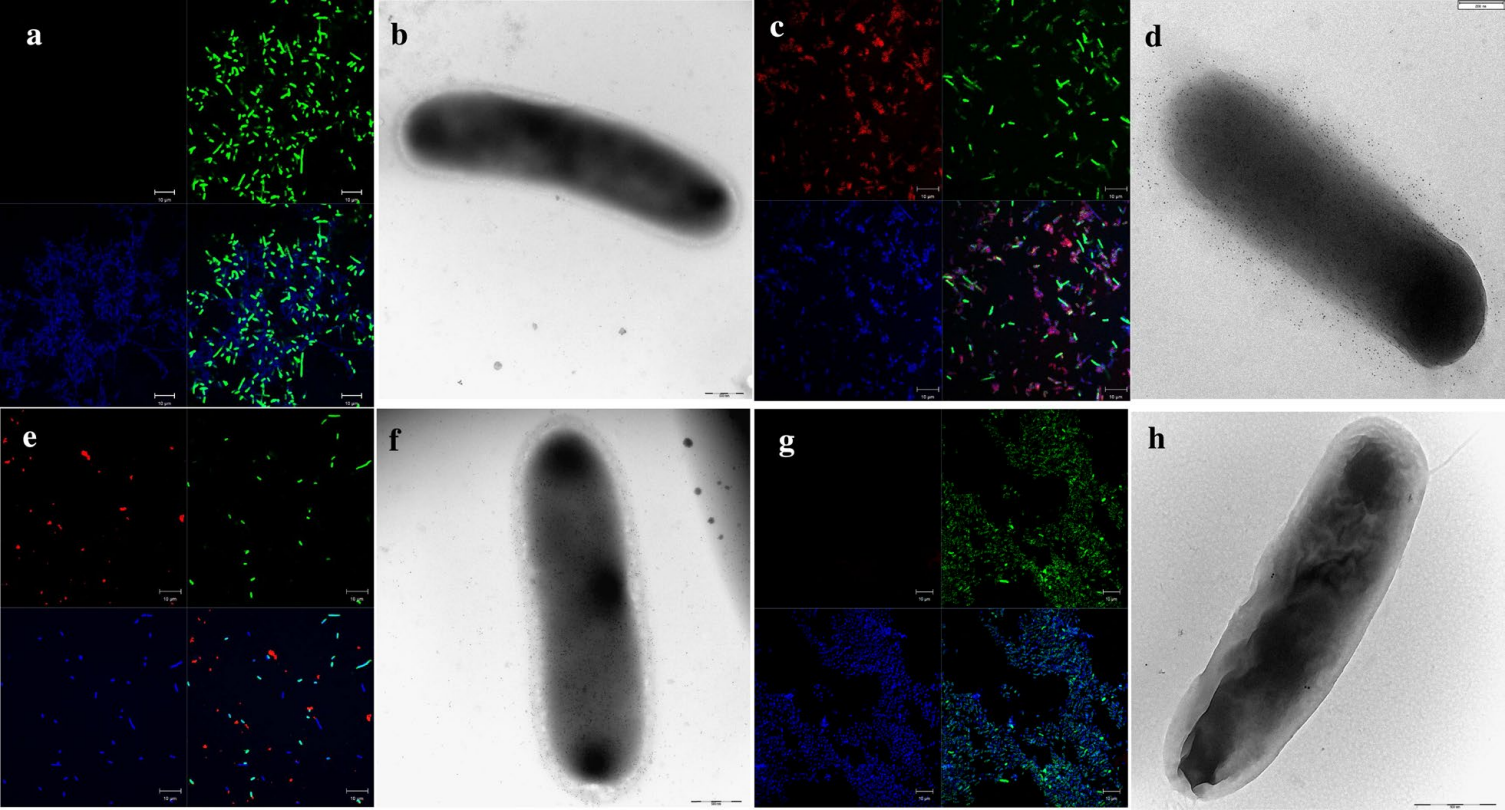
Confocal and Transmission electron microscopy of BamE expressed in *E. coli*. *E. coli* BL21(DE3) expressing the full-length neisserial BamE (**a** and **b**), or fused to AIDA-I (**c** and **d**), or the *E. coli* BamE fused to Lpp’OmpA (**e** and **f**) grown at 25 °C, were incubated first with anti-FLAG antibodies while *E. coli* T7ExpressIq (pET15b) expressing InaK fused to the neisserial BamE grown at 18 °C, was incubated first with polyclonal anti-*Nm*BamE antibodies (**g** and **h**). Subsequently samples were incubated with the secondary anti-mouse immunoglobulin G (whole molecule) Alexa fluor 568-conjugated. The lipoproteins can be visualized in red, the DNA in blue (DAPI) and the membranes in green (oregon green) (**a**, **c**, **e **and **f**). In transmission electron microscopy using immunogold labelling, the same samples were incubated with the secondary anti-mouse immunoglobulin G conjugated with 5 nm gold particles (**b**, **d**, **f** and **h**)

As expected, when not fused to a carrier protein, the full-length lipoprotein could not be visualised on the surface of the bacterium as was evident by i) the lack of red fluorescence signals in confocal microscopy (Fig. 6, panel A) and ii) the absence of gold particles in immunogold labelling with TEM (Fig. 6, panel B). When fused to AIDA-I, the neisserial BamE, which gave a positive FACS signal, was detected on the surface of almost all the bacterial cells observed by confocal microscopy (Fig. 6, panel C). The TEM analysis revealed that gold particles were localised on the entire surface of the bacterial cell which showed a well-preserved rod-like shape, thus indicating that the mature protein domain of interest was surface-exposed (Fig. 6, panel D). Hence, AIDA-I has proved to be an efficient delivery system, able to decorate the whole bacterial cell surface with the antigen expressed at a relatively high level. Confocal microscopy of the bacteria expressing the *Ec*-BamE lipoprotein fused to the Lpp’OmpA chimaera indicated that BamE was exposed on the surface of almost all bacterial cells (Fig. 6, panel E). The immunogold labelling showed a distribution of the gold particles over nearly the entire surface of the bacterial cell (Fig. 6, panel F), but to a lower extent compared to that observed for the AIDA-I construct. When the neisserial BamE is fused to INP, no signal associated with the protein of interest was observed with confocal microscopy and only very fewgold particles were present on the surface of the bacterial cell (Fig. 6, panels G and H, respectively). It should be noted that bacterial cells overexpressing *Nm*BamE fused to INP form large aggregates indicating that when the INP construct is overexpressed the bacterial outer membrane undergoes a dramatic change. This is suggested also by the uneven surface observed in the corresponding electron microscope image (Fig. 6, panel H). In addition, the Post-embedding Method using L.R. White Embedding Medium revealed the presence of aggregates of the overexpressed fusion protein even at 18 °C (Additional file 7: Figure S4).

## Discussion

In this work, AIDA-I, Lpp’OmpA and InaK-NC were selected as they have been previously described as efficient targeting vehicles for the surface localisation of a large number of soluble proteins [14, 15, 16, 17, 20, 21]. Nevertheless, there are few studies describing a direct comparison of the three analysed carrier proteins and they have been limited to only two of the systems, INP and the Lpp’OmpA chimaera. In particular, two research groups engineered *E. coli* by employing the Lpp’OmpA chimaera and different truncated forms of the ice nucleation protein (InaV) as anchoring motifs for the organophosphorus hydrolase (OPH) enzyme [46, 47]. In contrast to our study, the INP constructs successfully display OPH on the bacterial surface with good stability and functionality. However, as with our findings, loss of viability was observed in the engineered *E. coli* host strain [46, 47]. Furthemore, the effectiveness of each approach of surface dysplay has been always analysed by only one or few passenger proteins. Here, AIDA-I, Lpp’OmpA and InaK-NC were assessed in parallel as surface display systems for the exposure on the surface of *E. coli* of the same type of passenger protein: the mature portion of lipoproteins. The panel of investigated passenger proteins comprised eight lipoproteins: four lipoproteins of the host strain and four meningococcal lipoproteins from *N. meningitidis* strain NZ98/254.

Bacterial lipoproteins are membrane proteins characterised by a conserved lipid-modified cysteine residue at the N-terminus, which allows the mature protein domain to be anchored to a phospholipid in the lipid bilayer after secretion across the cell membrane and cleavage of the signal peptide. In Gram negatives, when the lipoproteins are not retained in the inner membrane (IM) the conserved Lol system can transport the lipoprotein across the periplasm to the outer membrane (OM) [30, 31,^32^]. Most lipoproteins are oriented towards the periplasm in the outer leaflet of the IM or in the inner leaflet of the OM, however, some lipoproteins have been reported to be surface exposed [48]. Homologues of the family of proteins known as surface lipoprotein assembly modulator, SLAM [49], have been identified as translocator proteins for a subset of surface lipoproteins which are substrates specific for the SLAM translocator, originally identified in *N. meningitidis* but present in a number of B-proteobacteria [49]. While Hooda and co-workers demonstrated that the co-expression in *E. coli* of SLAM1 from *N. meningitidis* with its specific lipoprotein substrate, fHbp, can lead to efficient surface exposure [49], another study reported that in *E. coli* the *N. meningitidis* fHbp spontaneously migrate to the cell surface in a functional conformation [50]. In our study all lipoproteins both of *E. coli* and *N. meningitidis* origin, when expressed as lipoproteins and in the absence of a delivery system, were not delivered to the surface of the outer membrane. However, most of the mature lipoprotein domains, when fused to the Lpp’OmpA chimaera or AIDA-I, were properly displayed on the surface of the host.

On the basis of our work, the most critical factors that determined the efficient delivery to the bacterial surface of the mature lipoproteins were found to be a lower induction temperature and/or specificity of the host strain system, which likely had the effect of optimisation of their expression leading to a balance between their rate of translation and secretion. In fact, as has already been reported, when the expression level is too high, the folding machinery and the trafficking systems could become overloaded, thus preventing the expressed protein from being efficiently translocated to the surface [40, 43, 51]. We found this to be especially true for the INP constructs, as demonstrated by the post-embedding TEM image that showed the presence of cytoplasmic aggregates of the overexpressed fusion protein, even at 18 °C (Additional file 7: Figure S4). It is important to recall that INP is a protein from *Pseudomonas syringae*, a plant pathogen bacterium only distantly related to *E. coli*. In fact, *P. syringae* is associated with frost damage to crops and commonly found living in the wider environment, including water sources and therefore at lower temperature [52]*.* INP exploits a targeting mechanism still not entirely understood with a rather particular anchoring of the protein based not only on a GPI anchor (a motif quite unique in prokaryotes) but also on N- and O- glycosylation [35]. At present, there is no consensus in the literature regarding the effectiveness of INP as a delivery system. It has been reported that the use of the ice nucleation protein for the display of heterologous proteins in *E. coli* depends on the degree of overexpression. At low expression levels, INP is translocated to the outer membranes, whereas in the case of high expression levels the protein is found mainly in inclusion bodies [22, 32, 34]. Nevertheless, it has also been demonstrated that the level of expression is not a critical factor. In particular, the growth temperature has been shown not to have an influence on the surface exposure of passenger proteins such as the green fluorescent protein GFP [35], the carbonic anhydrase from the thermophilic bacterium *Sulfurihydrogenibium yellowstonense* [20] and the human arginase-1 [21], enzymes that have been successfully exposed at 37 °C. In this work, a low growth temperature (18 °C) and the T7ExpressIq *E. coli* host strain were used in order to obtain a reduced basal level of expression. Despite all the optimisation attempts with the InaK-NC carrier protein, compared with the AIDA-I and Lpp’OmpA delivery systems, analogous levels of surface exposure of the lipoproteins used in this study were not achieved. Hence, our results have confirmed the concerns related to the effectiveness of INP for surface display applications in *E. coli*. Therefore, further work is needed to make this approach more generally applicable and reproducible. It would be interesting to evaluate other variants of the INP family, such as InaV and different truncated forms like the N-terminal domain alone or its combination with the C-terminal domain or the central repeating domain (CDR) [35, 54, 55]. An alternative strategy could be the use of different host strains, for example, *P. syringae, P. putida* or related species like *Moraxella spp* [56]. A better understanding of the INP translocation mechanisms is necessary to enable an optimisation of the experimental conditions necessary for this type of construct.

Lpp’OmpA is a good delivery system for surface exposure of homologous lipoproteins. In fact, all the host lipoproteins were surface exposed when fused to the Lpp’OmpA chimaera at all growth temperatures tested. However, cells from the same culture frequently showed two different populations in which a specific gene is either surface-exposed or not. This behaviour may be due to the occurrence of bistability [44, 45]. As possible consequence of this, the percentage of expressing cells showed a significant variation between cultures. In addition, in most cases viable bacteria represented only 64% of the bacterial population (Additional file 4: Figure S1, panel B). Hence, the difficulties in growing *E. coli* expressing this type of construct limited the use of Lpp’OmpA as a general surface display system. The level of expression had to be tightly regulated in order to avoid growth inhibition and phenotype heterogeneity. These observations are in agreement with previous reports [46, 47].

Within the context of our study, AIDA-I is clearly the most efficient delivery system for surface exposure of heterologous lipoproteins. Three of the four neisserial lipoproteins studied were efficiently exposed on the surface of BL21DE3 *E. coli* cells at both growth temperatures. In fact, expression of AIDA-I constructs could be easily obtained at 25 or 37 °C without affecting viability. It is clear from our data that the viability was higher compared with the other three types of construct (93 vs 60–64%, Additional file 4: Fig. S1). In addition, as demonstrated by confocal and electron microscopy, the passenger protein was expressed at high density on the surface of almost all the bacterial cells. In agreement with our results, this monomeric autotransporter has been previously described as an efficient delivery system for exporting a large number of proteins such as the *Salmonella* flagellar protein H:gm, the SE serotype-specific fimbrial protein SefA [14, 15] and subsequently their fusion product (H:gmdSefA) [16]. Interestingly, these epitopes appeared to be recognized by HT-29 intestinal cells, as determined by induction of the pro-inflammatory interleukin 8 [16]. Hence, the fusion proteins were in a functional conformation able to induce an immunogenic response.

### Conclusions

This is the first time that three surface display systems have been compared using a number of lipoprotein candidates. It has been demonstrated that the best delivery system to use cannot be defined a priori but has to be assessed case-by-case depending on the experimental conditions and the combination between the carrier and passenger proteins. Despite all the optimization steps performed, the truncated form InaK-NC did not allow an efficient surface exposure of the passenger proteins used in this study. However, we cannot exclude that other variants of the INP family, such as InaV and different truncated forms, may be more successful. In our experimental conditions, the Lpp’OmpA chimaera has proved to be an efficient surface display system for the homologous passenger proteins, but cell lysis and phenotype heterogeneity were observed. AIDA-I has been shown to be the best surface display system for mature lipoproteins (especially heterologous ones) in the *E. coli* host strain without growth inhibition and limited phenotype heterogeneity*.* A crucial aspect that is worthy of further investigation is the evaluation of the conformation and functionality of the constructs after delivery to the surface of *E. coli*. In fact, our study considered the translocation of a good number of mature lipoproteins in order to ascertain the characteristics of the most efficient system in more general terms. By contrary, in an elegant study of the efficiency of the autotransporter AIDA in translocating the enzyme tyrosinase across the bacterial membrane, the most favourable conditions were assessed by using the enzymatic activity of tyrosinase itself to monitor its correct translocation to the cell surface [57]. Therefore, a more thorough functional characterization of the displayed proteins in order to determine their antigenicity will constitute an important aspect of our future work.

## Methods

### Bacterial strains and plasmids

All the cloning steps have been carried out by using the PIPE (Polymerase Incomplete Primer Extension) method, a ligation-independent cloning technique [58, 59]. The list of the primers used in this work is presented in Additional file 1: Table S1. The first cloning step consisted in the insertion in the pET15b plasmid (Novagen) of the three delivery systems and as a negative control, the full-length genes encoding the analysed lipoproteins. The three display systems AIDA-I, Lpp’OmpA and InaK (N + C termini) have been synthesized as dsDNA fragments by GeneArt (Thermo Fisher Scientific, Additional file 2) and, subsequently, amplified by PCR to obtain the corresponding insert to be cloned between the T7 promoter and the T7 terminator. The newly generated expression plasmids were named pET15b::AIDA-I and pET15b::Lp’OmpA (Additional file 3: Table S2). In all constructs, the FLAG tag was fused to the C-terminus of the recombinant proteins to facilitate protein detection. The second cloning step was to insert the mature portion of each lipoprotein, predicted by the DOLOP software (https://www.mrc-lmb.cam.ac.uk/genomes/dolop/analysis.shtml), in frame with the delivery systems. The genomic DNA of *N. meningitidis* serogroup B (NZ98/254) and *E. coli* K-12 were used as templates for amplifying the coding regions of the lipoproteins of interest using Q5 DNA polymerase (Qiagen). All the unpurified PCR products (V-PCR and I-PCR) were used to directly transform chemically competent Mach1 T1R cells (Thermo Scientific). The screening of positive clones was performed by colony PCR and subsequently verified by sequencing. All expression experiments were performed using the BL21(DE3) (Thermo Scientific) and T7 express Iq (New England Biolabs) *E. coli* strains.

### Growth conditions

Bacteria were inoculated into Luria Bertani (LB) medium at 37 °C, 25 °C or 18 °C, with shaking at 160 rpm. When required, ampicillin was added to a final concentration of 100 μg/mL. Cultures grown overnight were diluted to give OD_600_ = 0.1 and when they reached OD_600_ = 0.6 expression of the recombinant fragment was induced with isopropyl β-d-1-thiogalactopyranoside (IPTG) (Sigma) at a final concentration of 1 mM. The time of induction was one/two hours.

### Gel electrophoresis and western blot analysis

A pellet corresponding to OD_600_ = 1.0 of each induced bacterial growth was resuspended in 50 μl of Cell Lytic (Sigma Aldrich), for 30′ at 37 °C in a thermomixer with shaking at 600–800 rpm. Total extracts were treated with Loading dye NuPage LDS Sample Buffer 4X (Thermo Scientific) and DTT 10X NuPage Reducing Agent (Thermo Scientific) and denatured at 95 °C for 5 min. Protein extracts were separated by SDS-PAGE on NuPAGE Novex 4–12% Bis–Tris Protein Gels in MES 1X (Thermo Scientific). Novex Sharp Pre-Stained Protein Standard (Thermo Scientific) was used as a molecular weight marker. Protein expression was evaluated by Western blot analysis**.** Protein extracts were transferred onto nitrocellulose membrane using an iBlot Dry Blotting System (Thermo Scientific). Membranes were saturated for 1 h at room temperature with PBS containing 0.05% (v/v) Tween 20 (Sigma) and 10% (w/v) milk Blotting-Grade Blocker (Biorad). Membranes were incubated at room temperature for one hour with mouse monoclonal ANTI-FLAG M2 antibody (Sigma), diluted 1:2000 in PBS with 1% (v/v) Tween 20 (Sigma) and 1% milk. After being rinsed three times with PBS to remove non-specific binding (10 min each), membranes were incubated for one hour with horseradish peroxidase (HRP)-conjugated anti-mouse IgG antibody (DAkO), diluted (1:2000) in PBS + 1% (v/v) Tween 20 (Sigma) and 1% (w/v) powdered milk (Sigma). Membranes were then again washed three times with PBS. The Biorad OPTI-4CN substrate kit was used according to the manufacturer’s instructions.

### Labelling for FACS analysis

Approximately 10^8^ bacteria were collected by centrifugation (10,000 g for 5 min). Bacteria were fixed for 15 min at 4 °C with PBS containing 2% (v/v) formaldehyde (Sigma). The fixed bacteria were then suspended in PBS containing 1% BSA (w/v) for 16–24 h at 4 °C. Inactivated bacteria were centrifuged, resuspended in 100 µL of a solution containing monoclonal ANTI-FLAG M2 antibody produced in mouse (or the specific mouse polyclonal sera anti-CsgG and anti-NmBamE) diluted 1:500 in PBS containing 1% BSA and incubated 1 h at room temperature. Bacteria were washed with 500 µL of PBS + 1% BSA. Each bacterial pellet was then resuspended in 100 µL of a secondary rabbit anti-mouse FITC-conjugated immunoglobulin G (whole molecule) (Sigma) diluted 1:250 in PBS + 1% BSA and incubated for 1 h in the dark. After a final washing step, the cells were resuspended in 200 μL of PBS. All data were collected using a BD FACS CANTO II (BD Bioscience) by acquiring 10,000 events, and the data analysed using the Flow-Jo software (v.8.6, TreeStar Inc). The combination of the morphologic gate (x = FSC-A and y = SSC-A) and single gate (x = SSC-W and y = SSC-A) ensures that only viable and single bacterial cells which do not form aggregates are considered.

### Labelling for Immunofluorescence analysis

Strains were grown as described in the section “Growth conditions”. Approximately 10^8^ bacterial cells were collected by centrifugation (10,000×g for 5 min). Bacteria were washed with 300 µL of PBS and fluorescently labelled with Oregon Green 488 Carboxylic Acid, Succinimidyl Ester, 6-isomer (Thermo Scientific) diluted 1:1000 in PBS. Bacteria were washed twice with 300 µL of sterile PBS and then resuspended in 100 µL of PBS containing 2% (v/v) formaldehyde (Sigma). Samples were spotted onto a POLYSINE slide (Menzel-Glaser) and incubated for 10–15 min. Bacteria were washed with 100 µL of PBS and then incubated for 40 min at room temperature with the monoclonal ANTI-FLAG M2 antibody produced in mouse (Sigma) mAb diluted 1:500. Bacteria were washed with 300 µL of PBS and incubated for 20–30 min at RT in the dark with 100 µL of PBS containing a secondary rabbit anti-mouse immunoglobulin G (whole molecule) Alexa fluor 568-conjugated (Thermo Scientific) diluted 1:250. After two washes with PBS, a droplet of a mounting solution containing DAPI was applied. The final step consisted in placing a cover glass on each spot and analysing the samples with a confocal ZEISS LSM700 microscope.

### Labelling for transmission electron microscopy

Strains were grown as previously described. Approximately 2 × 10^9^ bacterial cells were resuspended in 1 mL of PBS and fixed with 4% paraformaldehyde. 5 µL of each sample were applied to a 200-square mesh nickel grid coated with a thin carbon film. Samples were blocked with PBS + 1% BSA and then incubated for 1 h at RT with the primary antibody (diluted 1:250 in the blocking solution). Grids were washed twice and incubated with gold labelled anti-mouse secondary antibodies (diluted 1:40 in 1% PBS-BSA) for 1 h. Samples were washed in distilled water and observed using TEM FEI Tecnai G2 Spirit operating at 100 kV and equipped with a CCD Olympus SIS Morada camera (Olympus, Shinjuku, Tokyo, Japan). Images were acquired and processed using the iTemm (OSIS, Olympus, Shinjuku, Tokyo, Japan) software.

To verify the presence of aggregates in T7expressIq (pET15b)INP-*Nm*BamE, post-embedding experiments were performed. The sample was divided into aliquots and fixed O/N at 4 °C in 0.1 M sodium cacodylate buffer containing 2.5% glutaraldehyde and 2.5% formaldehyde and then post-fixed in 1% OsO_4_. Samples were then dried by the critical point method using CO_2_ in a Balzers Union CPD 020. The dried samples were embedded in LRWhite resin and stained with uranyl acetate and lead citrate.

## Supplementary Information

**Additional file 1: Table S1.** List of primers used in this study.

**Additional file 2.** DNA sequence for the delivery systems used in this study.

**Additional file 3: Table S2.** List of expression plasmids used in this study.

**Additional file 4: Figure S1.** FACS analysis of viable and not aggregated bacteria. For each of the four engineered constructs, a representative example was displayed (y= SSC-A and x= SSC-W). A) Full-length lipoprotein and its fused forms with B) AIDA-I, C) Lpp’OmpA and D) InaK.

**Additional file 5: Figure S2.** Expression of InaK fusion proteins in E. coli, at 37°C and 25°C. A) SDS-PAGE of whole-cell lysates showing inaK fusion proteins, at 25°C. Lane 1: Marker, Lane 2: CsgG, Lane 3: NmBamE, Lane 4: putative lipoprotein, Lane 5: LolB, Lane 6: BamE, Lane 7: LptE, Lane 8: Pal and Lane 9: NmMtrC. FACS analysis of InaK fusion proteins in E. coli, at 37°C and 25°C . E. coli BL21DE3 (pET15b) expressing InaK fused the N. meningitidis lipoproteins: CsgG, MtrC, BamE and putative lipoprotein at 37°C (B) and 25°C (D) and the E. coli lipoproteins: LolB, LptE, Pal and BamE at 37°C (C) and 25°C (E) were incubated with monoclonal anti-FLAG antibody. The grey areas represent the fluorescence signals obtained with the control (BL21DE3-pET15b ∅). The coloured lines represent the fused forms of the lipoproteins. Panels B and D (heterologous lipoproteins): Purple: CsgG, Dark Red: MtrC, Light Blue: nmBamE, Dark Green: putative lipoprotein; Panels C and E (homologous lipoproteins): Dark Blue: BamE, Light Green: LolB, Orange: LptE, Light Red: Pal.

**Additional file 6: Figure S3.** FACS analysis of NmBamE fused to the InaK delivery system in E. coli, at 25°C using the polyclonal anti-NmBamE antibodies. E. coli BL21DE3 and E. coli T7ExpressIq (pET15b) expressing InaK fused the N. meningitidis lipoprotein BamE at 25°C (A and B, respectively) were incubated with the polyclonal anti-NmBamE antibodies. The grey areas represent the fluorescence signals obtained with the control (BL21DE3-pET15b ∅ or T7ExpressIq pET15b ∅, panels A and B, respectively). The Light Blue coloured line represents the fused forms of the NmBamE lipoprotein.

**Additional file 7: Figure S4.** The Post-embedding Method using L.R. White Embedding Medium was performed to verify the presence of aggregates in T7expressIq (pET15b) InaK-NmBamE.

## Data Availability

The datasets used and analysed during the current study are available from the corresponding author.

## Abbreviations

AIDA-I: Adhesin involved in diffuse adherence-I
AT: Autotransporter
Bam: β-Barrel assembly machinery
BSA: Bovine serum albumin
CRD: Central repeating domains
DAPI: 4′,6-Diamidino- 2 phenylindole
EPEC: Enteropathogenic *E. coli*
FACS: Fluorescence activated cell sorting
FITC: Fluorescein isothiocyanate
FSC-A: Forward Scatter Area
GC: Guanine-cytosine
GPI: Glycosylphosphatidylinositol
His-tag: Histidine tag
HRP: Horseradish peroxidase
IM: Inner membrane
IPTG: Isopropyl β-d-1-thiogalactopyranoside
INP: Ice nucleation protein
LB: Luria Bertani
Lgt: Diacylglyceryl transferase
Lnt: N-acyltransferase
Lol: Localisation of lipoprotein
Lpp: Braun's lipoprotein
LspA: Signal peptidase II
M.W.: Molecular weight
MBP: Maltose binding protein
MES: 2-(N-morpholino) ethanesulfonic acid
OD: Optical density
OM: Outer membrane
OmpA: Outer membrane protein A
OMPs: Outer membrane proteins
OPH: Organophosphorus hydrolase
PBS: Phosphate-buffered saline
PIPE: Polymerase incomplete primer extension
PMSF: Phenylmethhylsulfonyl fluoride
RT: Room temperature
SDS-PAGE: Sodium dodecyl sulfate Polyacrylamide gel electrophoresis
SSC-A: Side Scatter Area
SSC-W: Side Scatter Width
TAM: Translocation and Assembly Module
T5SS: Type V secretion system
TEM: Transmission electron microscope
TEV: Tobacco etch virus
