# Supplementary material for: Decorating the surface of Escherichia coli with bacterial lipoproteins: a comparative analysis of different display systems
Source: Microb Cell Fact. 2021 Feb 2;20:33. doi: 10.1186/s12934-021-01528-z (PMC7853708; doi:10.1186/s12934-021-01528-z)
Supplement: Supplementary file 2 — Additional file 2. DNA sequence for the delivery systems used in this study. [file 12934_2021_1528_MOESM2_ESM.docx]

**>AIDA-I**

atgaacaaagcctacagcatcatttggagccatagccgtcaggcatggattgttgcaagcgaactggcacgtggtcatggttttgttctggcaaaaaataccctgctggttctggcagttgtttcaaccattggtaatgcattcgccgattataaggatgacgatgacaaagactacaaagatgatgatgataaagagaacctgtactttcaaggtggtcgtcgtaccagcgcaggtaataccctgaccgttagcaattacaccggtacaccgggtagcgttattagcttaggtggtgttctggaaggtgataattcactgaccgatcgtctggttgttaaaggcaataccagcggtcagagcgatattgtttatgtgaatgaagatggtagcggtggtcagacccgtgatggtattaacattattagcgtggaaggtaactccgatgcagaatttagcctgaaaaatcgtgttgttgccggtgcatatgattataccctgcagaaaggtaatgaaagcggcaccgataataaaggttggtatctgaccagccatctgccgaccagcgatacccgtcagtatcgtccggaaaatggtagctatgcaaccaatatggcactggcaaatagcctgtttctgatggatctgaatgaacgtaaacagtttcgtgccatgagcgataatacccagccggaaagcgcaagcgtttggatgaaaatcaccggtggtattagcagcggtaaactgaatgatggtcagaataaaaccaccaccaaccagtttattaaccagttaggtggcgacatctacaaatttcatgcagaacagctgggtgatttcaccctgggcattatgggtggttatgcaaatgcaaaaggcaagaccattaactacaccagcaataaagcagcacgcaataccctggatggttatagcgttggtgtttatggcacctggtatcagaatggtgaaaatgcaaccggtctgtttgcagaaacctggatgcagtataattggtttaatgccagcgttaaaggtgatggtctggaagaagaaaaatacaatctgaatggtctgaccgcaagtgcaggcggtggctataacctgaatgttcatacctggaccagtccggaaggtattaccggtgaattttggctgcaaccgcatctgcaggcagtttggatgggtgtgacaccggatacacatcaagaagataacggcaccgttgttcaaggtgcaggtaaaaacaatattcagaccaaagcaggtattcgtgcaagctggaaagttaaaagcaccctggataaagataccggtcgtcgttttcgtccgtatattgaagcaaattggatccataacacccacgaatttggtgtgaaaatgagtgatgatagccagctgctgagcggtagccgtaatcagggcgaaatcaaaaccggtattgaaggtgttattacccagaatctgagcgttaatggtggtgttgcatatcaggcaggcggtcatggtagcaatgccattagcggtgcactgggtatcaaatatagcttt

**>Lpp’OmpA**

atgaaagctactaaactggtactgggcgcggtaatcctgggttctactctgctggcaggttgctccagcaacgctaaaatcgatcagaacaacaatggcccgacccatgaaaaccaactgggcgctggtgcttttggtggttaccaggttaacccgtatgttggctttgaaatgggttacgactggttaggtcgtatgccgtacaaaggcagcgttgaaaacggtgcatacaaagctcagggcgttcaactgaccgctaaactgggttacccaatcactgacgacctggacatctacactcgtctgggtggcatggtatggcgtgcagacactaaatccaacgtttatggtaaaaaccacgacaccggcgtttctccggtcttcgctggcggtgttgagtacgcgatcactcctgaaatcgctacccgtgactacaaggacgacgatgacaagtgatag

**> InaK**

atgactctcgacaaggcgttggtgctgcgtacctgtgcaaataacatggccgatcactgcggccttatatggcccgcgtccggcacggtggaatccagatactggcagtcaaccaggcggcatgagaatggtctggtcggtttactgtggggcgctggaaccagcgcttttctaagcgtgcatgccgatgctcgatggattgtctgtgaagttgccgttgcagacatcatcagtctggaagagccgggaatggtcaagtttccgcgggccgaggtggttcatgtcggcgacaggatcagcgcgtcacacttcatttcggcacgtcaggccgaccctgcgtcaacgtcaacgtcaacgtcaacgtcaacgttaacgccaatgcctacggccatacccacgcccatgcctgcggtagcaagtgtcacgttaccggtggccgaacaggcccgtcatgaagtgttcgatgtcgcgtcggtcagcgcggctgccgccccagtaaacaccctgccggtgacgacgccgcagaatttgcagaccggatccagactctgggacgggaagaggtacaggcaactggtcgccagaacgggtgagaacggtgttgaggccgacataccgtattacgtgaacgaagatgacgatattgtcgataaacccgacgaggacgatgactggatagaggtaaaggactacaaggacgacgatgacaagtga
