# Supplementary figures and images for: Decorating the surface of Escherichia coli with bacterial lipoproteins: a comparative analysis of different display systems
Source: Microb Cell Fact. 2021 Feb 2;20:33. doi: 10.1186/s12934-021-01528-z (PMC7853708; doi:10.1186/s12934-021-01528-z)

## Slide 1
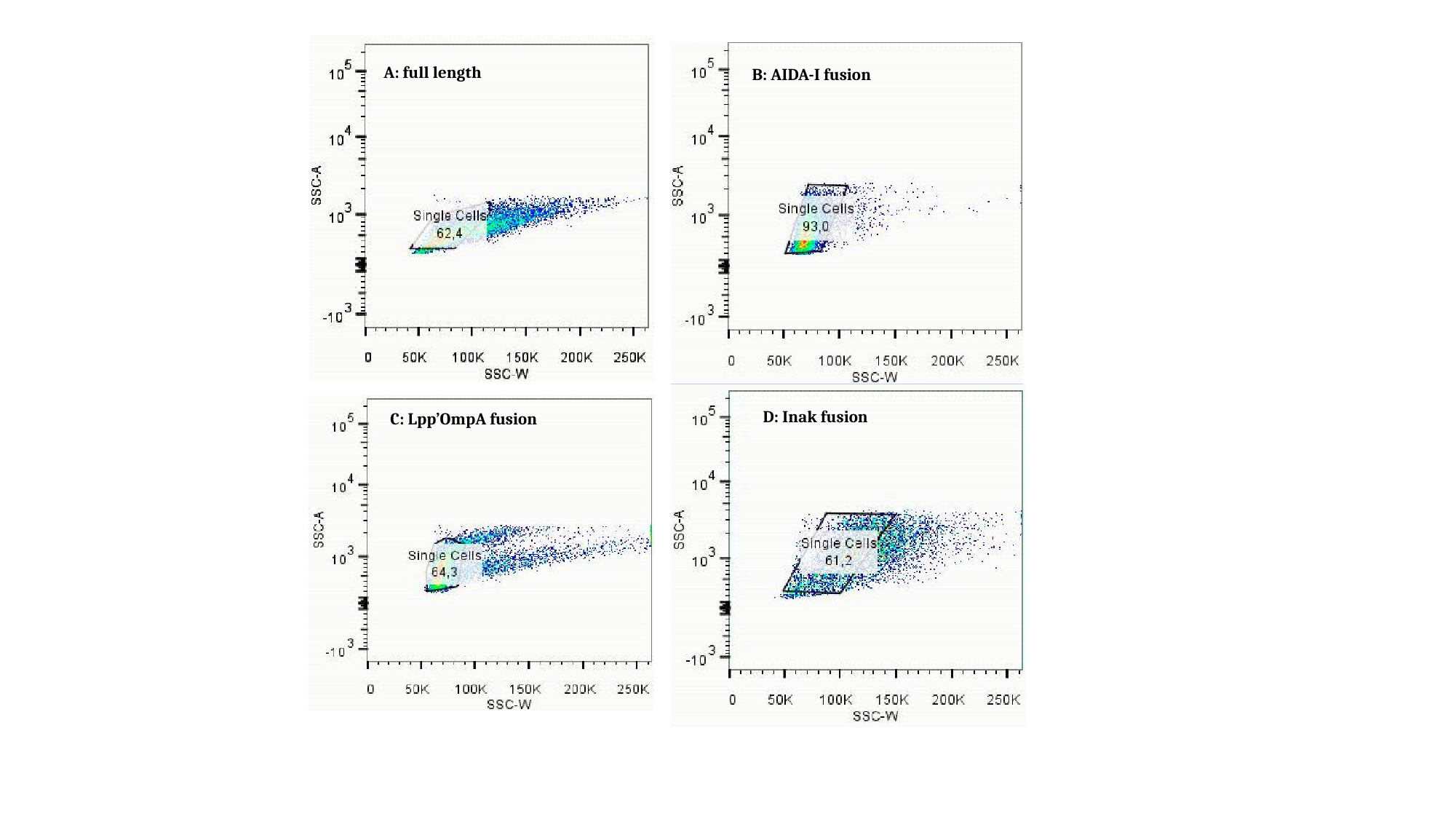

A: full length
B: AIDA-I fusion
D: Inak fusion
C: Lpp’OmpA fusion

Supplement: Supplementary file 4 — Additional file 4: Figure S1. FACS analysis of viable and not aggregated bacteria. For each of the four engineered constructs, a representative example was displayed (y= SSC-A and x= SSC-W). A) Full-length lipoprotein and its fused forms with B) AIDA-I, C) Lpp’OmpA and D) InaK. [file 12934_2021_1528_MOESM4_ESM.pptx]

## Slide 1
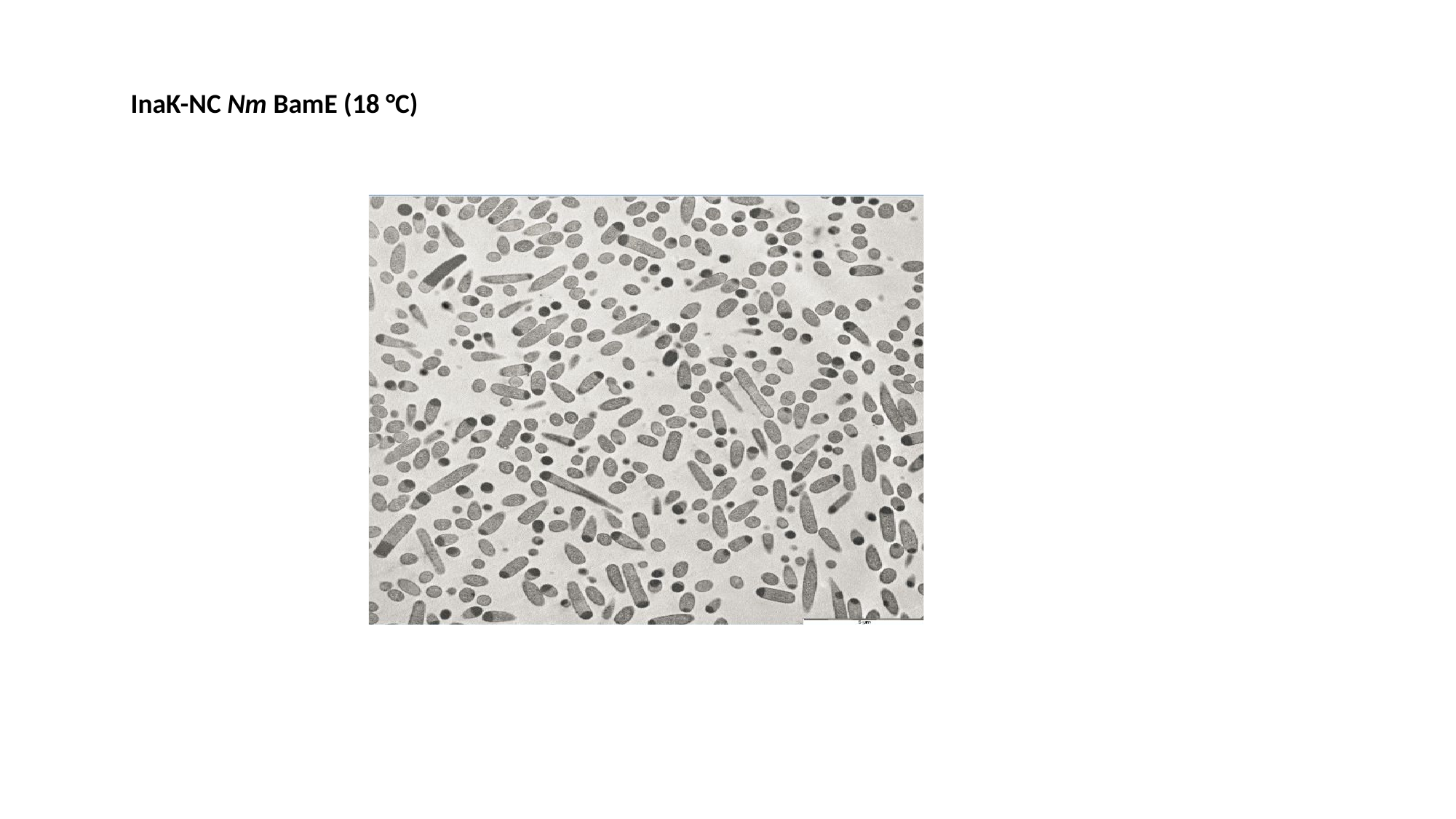

InaK-NC Nm BamE (18 °C)

Supplement: Supplementary file 7 — Additional file 7: Figure S4. The Post-embedding Method using L.R. White Embedding Medium was performed to verify the presence of aggregates in T7expressIq (pET15b) InaK-NmBamE. [file 12934_2021_1528_MOESM7_ESM.pptx]
