# Supplementary material for: Decorating the surface of Escherichia coli with bacterial lipoproteins: a comparative analysis of different display systems
Source: Microb Cell Fact. 2021 Feb 2;20:33. doi: 10.1186/s12934-021-01528-z (PMC7853708; doi:10.1186/s12934-021-01528-z)
Supplement: Supplementary file 5 — Additional file 5: Figure S2. Expression of InaK fusion proteins in E. coli, at 37°C and 25°C. A) SDS-PAGE of whole-cell lysates showing inaK fusion proteins, at 25°C. Lane 1: Marker, Lane 2: CsgG, Lane 3: NmBamE, Lane 4: putative lipoprotein, Lane 5: LolB, Lane 6: BamE, Lane 7: LptE, Lane 8: Pal and Lane 9: NmMtrC. FACS analysis of InaK fusion proteins in E. coli, at 37°C and 25°C . E. coli BL21DE3 (pET15b) expressing InaK fused the N. meningitidis lipoproteins: CsgG, MtrC, BamE and putative lipoprotein at 37°C (B) and 25°C (D) and the E. coli lipoproteins: LolB, LptE, Pal and BamE at 37°C (C) and 25°C (E) were incubated with monoclonal anti-FLAG antibody. The grey areas represent the fluorescence signals obtained with the control (BL21DE3-pET15b ∅). The coloured lines represent the fused forms of the lipoproteins. Panels B and D (heterologous lipoproteins): Purple: CsgG, Dark Red: MtrC, Light Blue: nmBamE, Dark Green: putative lipoprotein; Panels C and E (homologous lipoproteins): Dark Blue: BamE, Light Green: LolB, Orange: LptE, Light Red: Pal. [file 12934_2021_1528_MOESM5_ESM.pptx]

## Slide 1
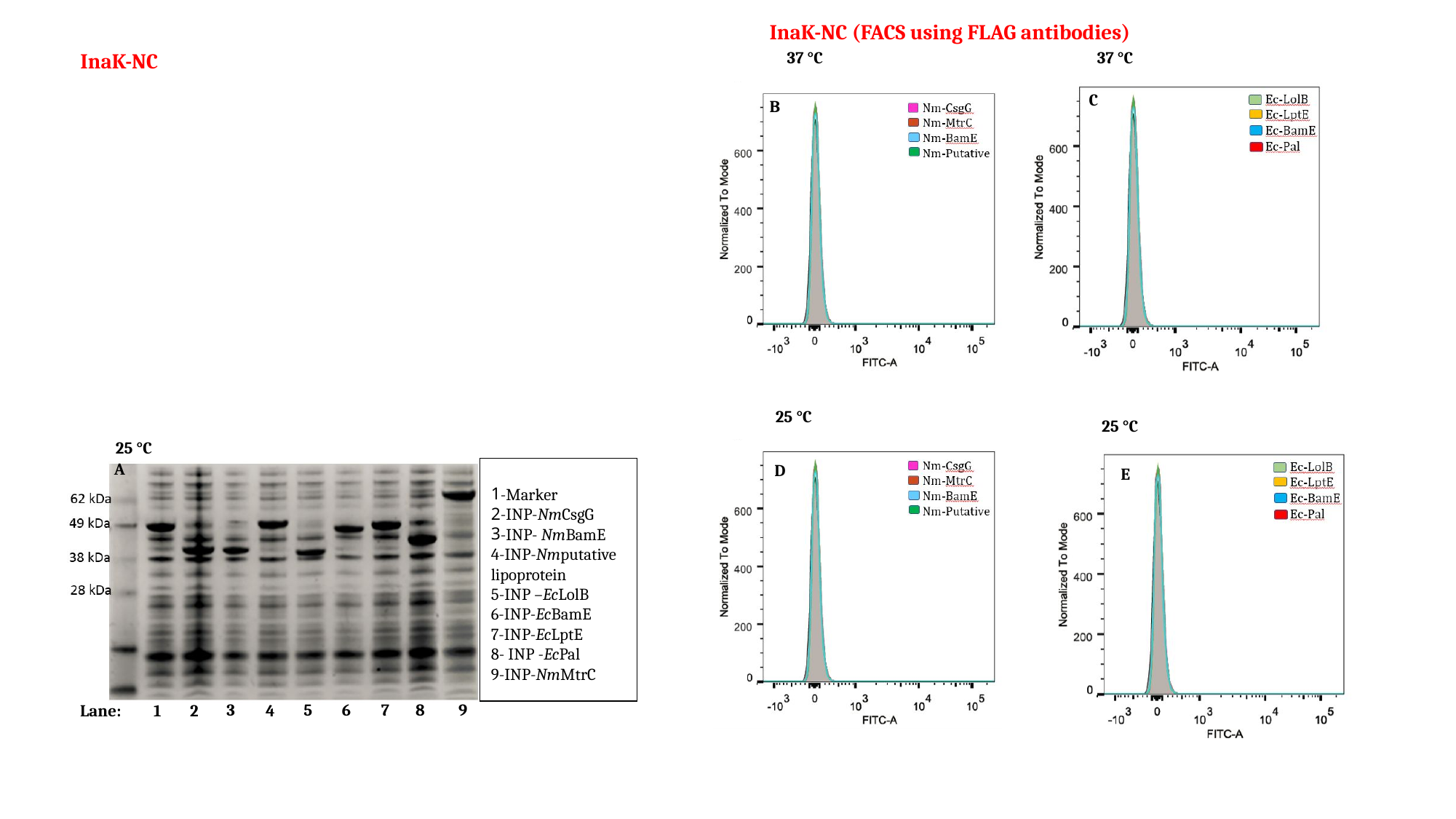

InaK-NC (FACS using FLAG antibodies)
37 °C
37 °C
InaK-NC
C
B
25 °C
25 °C
25 °C
A
D
-Marker
-INP-NmCsgG
-INP- NmBamE
4-INP-Nmputative lipoprotein
5-INP –EcLolB
6-INP-EcBamE
7-INP-EcLptE
8- INP -EcPal
9-INP-NmMtrC
E
3
5
6
7
8
9
Lane:
1
2
4
