# Supplementary material for: Decorating the surface of Escherichia coli with bacterial lipoproteins: a comparative analysis of different display systems
Source: Microb Cell Fact. 2021 Feb 2;20:33. doi: 10.1186/s12934-021-01528-z (PMC7853708; doi:10.1186/s12934-021-01528-z)
Supplement: Supplementary file 6 — Additional file 6: Figure S3. FACS analysis of NmBamE fused to the InaK delivery system in E. coli, at 25°C using the polyclonal anti-NmBamE antibodies. E. coli BL21DE3 and E. coli T7ExpressIq (pET15b) expressing InaK fused the N. meningitidis lipoprotein BamE at 25°C (A and B, respectively) were incubated with the polyclonal anti-NmBamE antibodies. The grey areas represent the fluorescence signals obtained with the control (BL21DE3-pET15b ∅ or T7ExpressIq pET15b ∅, panels A and B, respectively). The Light Blue coloured line represents the fused forms of the NmBamE lipoprotein. [file 12934_2021_1528_MOESM6_ESM.pptx]

## Slide 1
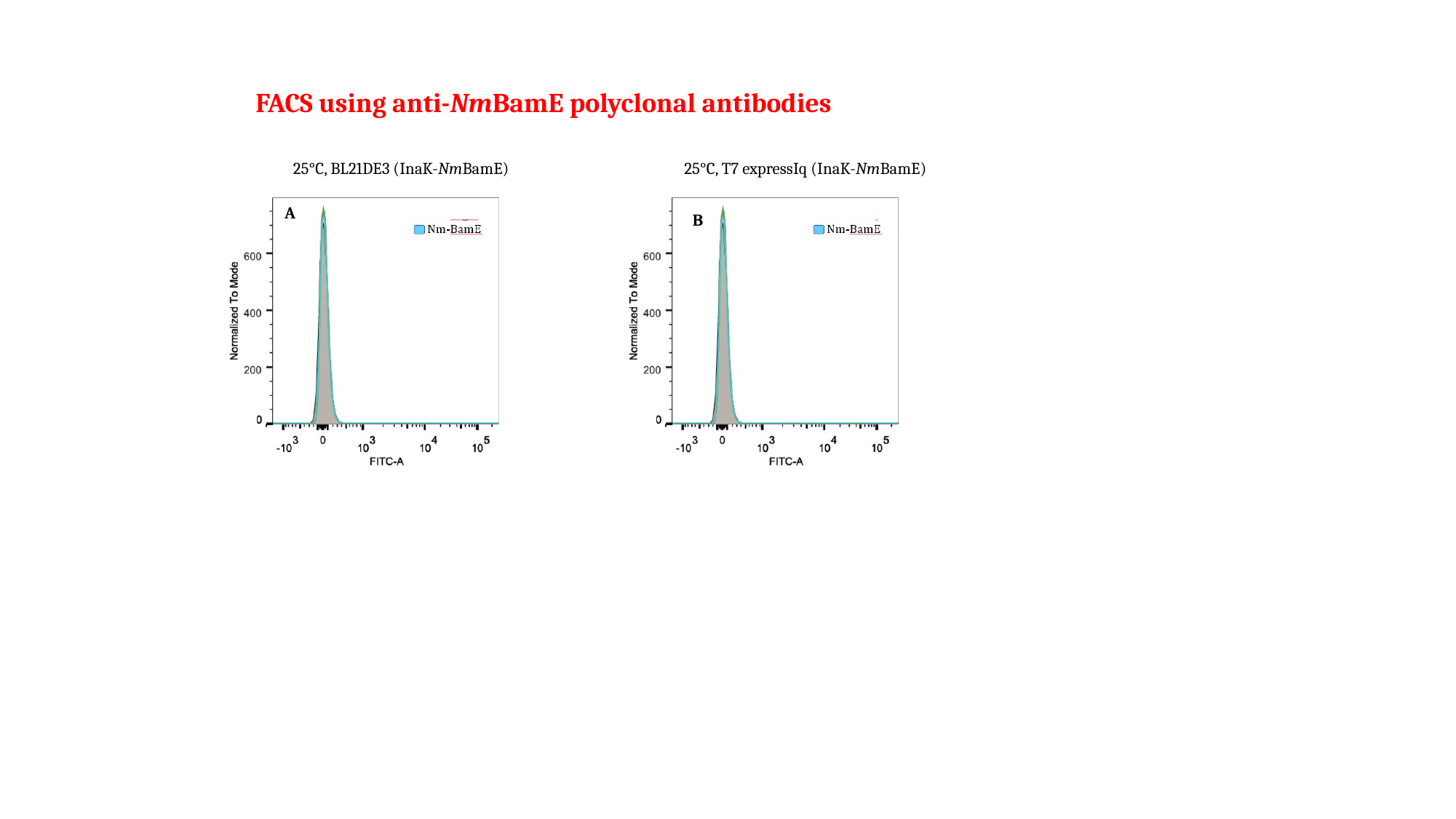

FACS using anti-NmBamE polyclonal antibodies
25°C, BL21DE3 (InaK-NmBamE)
25°C, T7 expressIq (InaK-NmBamE)
A
B
